# 10α-Hy­droxy-13-{[4-(2-hy­droxy­phen­yl)piperazin-1-yl]meth­yl}-4,9-dimethyl-3,8,15-trioxatetra­cyclo­[10.3.0.0^2,4^.0^7,9^]penta­decan-14-one

**DOI:** 10.1107/S1600536812013876

**Published:** 2012-04-06

**Authors:** Mohamed Moumou, Ahmed Benharref, Lahcen El Ammari, Mina Adil, Moha Berraho

**Affiliations:** a Laboratoire de Chimie Bioorganique et Analytique, URAC 22, BP 146, FSTM, Université Hassan II, Mohammedia–Casablanca 20810 Mohammedia, Morocco; b Laboratoire de Chimie Biomoléculaire, Substances Naturelles et Réactivité, URAC 16, Faculté des Sciences Semlalia, BP 2390, Bd My Abdellah, 40000 Marrakech, Morocco; c Laboratoire de Chimie du Solide Appliquée, Faculté des Sciences, Avenue Ibn Battouta, BP 1014 Rabat, Morocco

## Abstract

The title compound, C_25_H_34_N_2_O_6_, was synthesized from 9α-hy­droxy­parthenolide (9α-hy­droxy-4,8-dimethyl-12-methyl­ene-3,14-dioxatricyclo­[9.3.0.0^2,4^]tetra­dec-7-en-13-one), which was isolated from the chloro­form extract of the aerial parts of *Anvillea radiata*. The ten-membered ring adopts an approximate chair–chair conformation, while the piperazine ring displays a near regular chair conformation and the five-membered ring an envelope conformation with the C atom closest to the hy­droxy group forming the flap. The mol­ecular conformation is stabilized by an O—H⋯N hydrogen bond, which generates an *S*(7) loop, and the crystal structure features weak C—H⋯O inter­actions.

## Related literature

For background to the medicinal uses of the plant *Anvillea adiata*, see: Abdel Sattar *et al.* (1996 ▶); El Hassany *et al.* (2004 ▶); Qureshi *et al.*(1990 ▶). For the reactivity of this sesquiterpene, see: Hwang *et al.* (2006 ▶); Neukirch *et al.* (2003 ▶); Neelakantan *et al.* (2009 ▶). For ring puckering parameters, see: Cremer & Pople (1975 ▶). For the synthesis, see: Moumou *et al.* (2010 ▶).
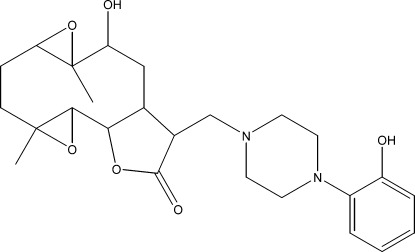


## Experimental

### 

#### Crystal data

C_25_H_34_N_2_O_6_
*M* *_r_* = 458.54
Orthorhombic, 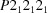

*a* = 8.0978 (2) Å
*b* = 10.3660 (3) Å
*c* = 28.8194 (8) Å
*V* = 2419.15 (11) Å^3^
*Z* = 4
Mo *K*α radiation
μ = 0.09 mm^−1^
*T* = 180 K
0.27 × 0.21 × 0.06 mm

#### Data collection

Agilent Xcalibur Sapphire1 long-nozzle diffractometer
27441 measured reflections
2829 independent reflections
2540 reflections with *I* > 2σ(*I*)
*R*_int_ = 0.034

#### Refinement

*R*[*F* ^2^ > 2σ(*F* ^2^)] = 0.034
*wR*(*F* ^2^) = 0.093
*S* = 1.05
2829 reflections
302 parameters
H-atom parameters constrained
Δρ_max_ = 0.19 e Å^−3^
Δρ_min_ = −0.18 e Å^−3^

### 

Data collection: *CrysAlis PRO* (Agilent, 2010 ▶); cell refinement: *CrysAlis PRO*; data reduction: *CrysAlis PRO*; program(s) used to solve structure: *SHELXS97* (Sheldrick, 2008 ▶); program(s) used to refine structure: *SHELXL97* (Sheldrick, 2008 ▶); molecular graphics: *ORTEP-3 for Windows* (Farrugia, 1997 ▶) and *PLATON* (Spek, 2009 ▶); software used to prepare material for publication: *WinGX* (Farrugia, 1999 ▶).

## Supplementary Material

Crystal structure: contains datablock(s) I, global. DOI: 10.1107/S1600536812013876/bt5864sup1.cif

Structure factors: contains datablock(s) I. DOI: 10.1107/S1600536812013876/bt5864Isup2.hkl

Supplementary material file. DOI: 10.1107/S1600536812013876/bt5864Isup3.cml

Additional supplementary materials:  crystallographic information; 3D view; checkCIF report

## Figures and Tables

**Table 1:** Hydrogen-bond geometry (Å, °)

*D*—H⋯*A*	*D*—H	H⋯*A*	*D*⋯*A*	*D*—H⋯*A*
O5—H5⋯N1	0.82	2.15	2.952 (2)	164
C1—H1⋯O4^i^	0.98	2.49	3.440 (3)	165
C10—H10⋯O1^ii^	0.98	2.36	3.232 (2)	148
C18—H18*A*⋯O6	0.97	2.32	2.943 (3)	121
C25—H25⋯O2^iii^	0.93	2.55	3.299 (4)	138

Symmetry codes: (i) 
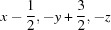
; (ii) 
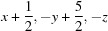
; (iii) 
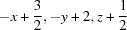
.

**Fig. 1.**
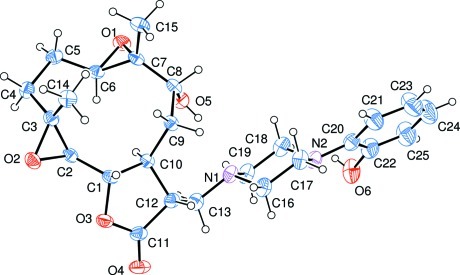
: Molecular structure of the title compound with displacement ellipsoids drawn at the 30% probability level. H atoms are represented as small spheres of arbitrary radii.

**Fig. 2.**
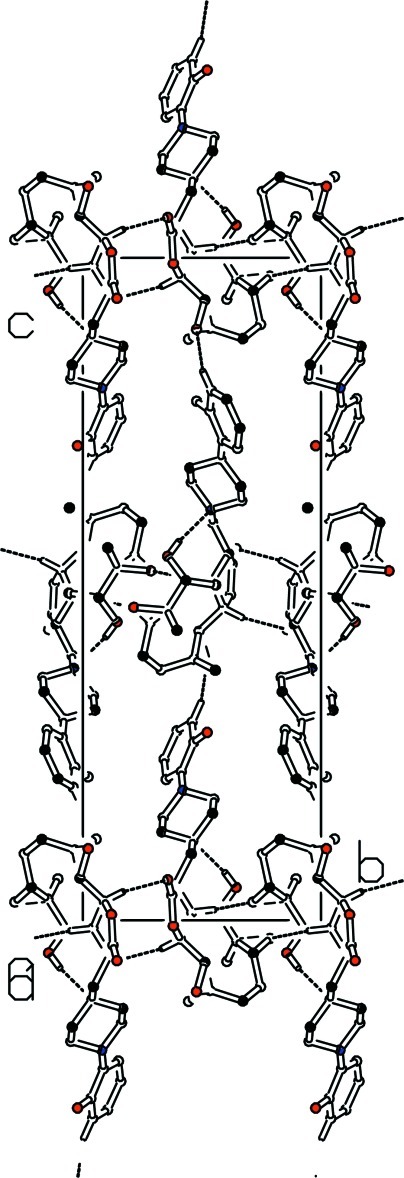
: Partial packing view showing the C–H···O hydrogen bonds as dashed lines. H atoms not involved in hydrogen bonding have been omitted for clarity.

## supplementary crystallographic information

### Comment

The natural sesquiterpene lactone, 9α - hydroxypartenolide is the main constituent of the chloroform extract of the aerial parts of *Anvillea radiata* (El Hassany *et al.*, 2004) and of *Anvillea garcini* (Abdel Sattar *et al.* (1996). The reactivity of this sesquiterpene lactone and its derivatives have been the subject of several studies Neukirch *et al.*, 2003; Hwang *et al.*, 2006; Neelakantan *et al.*, 2009), in order to prepare products with high value which can be used in the pharmacological industry. In this context, we have synthesed, from 9α-hydroxyparthenolide, the 6β,7α-epoxy-9α- hydoxypartenolide (9α-hydroxy-4,8-dimethyl-12-methylen-3,14-dioxa-tetracyclo [9.3.0.0^2^,^4^]tetradec-7-en-13-one)(Moumou *et al.*, 2010). This Epoxy-hydroxypartenolide treated with one equivalent of 1-(2-hydroxyphenyl- piperazine) gives the title compound(I)with a yield of 93%. The crystal structure of (I) is reported herein.The molecule contains a fused ring system and hydroxyphenylpiperazine group as a substituent to a lactone ring. The molecular structure of (I), Fig.1, shows the lactone ring to adopt an envelope conformation, as indicated by Cremer & Pople (1975) puckering parameters Q = 0.306 (2) Å and φ =79.5 (4)°. The ten-membered ring displays an approximate chair-chair conformation, while the piperazine ring has a perfect chair conformation with QT = 0.582 (3) Å, θ = 180.0 (2)° and φ2 =319 (24)°. In the crystal, C—H···O hydrogen bonding links the molecules into sheets lying parallel to the *bc* plane (Table 1, Fig.2). In addition an intramolecular O—H···N hydrogen bond is also observed.

### Experimental

The mixture of 6β,7α-epoxy-9α-hydoxypartenolide(9α-hydroxy- 4,8-dimethyl-12-methylen-3,14-tricyclo [9.3.0.0_2_,_4_]tetradec-7-en-13-one) (5 g, 3.57 mmol) and one equivalent of 1-(2-Hydroxyphenyl-piperazine) in EtOH (30 ml)was stirred for twelve hours at room temperature. Then the reaction was stopped by adding water (20 ml) and extracted three times with ethyl acetate (3 *x* 30 ml). The combined organic layers were dried over anhydrous MgSO_4_, filtered and concentrated under vacuum to give 1.5 g (3.32 mmol) of the title compound, which was recrystalized in ethyl acetate.

### Refinement

Reflections (0 0 2) and (0 1 1) were obstructed by the beam stop and were omitted from the refinement. All H atoms were fixed geometrically and treated as riding with C—H = 0.96 Å (methyl), 0.97 Å (methylene), 0.98 Å (methine) with *U*_iso_(H) = 1.2*U*_eq_(methylene, methine) or *U*_iso_(H) = 1.5*U*_eq_(methyl, OH). In the absence of significant anomalous scattering, the absolute configuration could not be reliably determined and thus Friedel pairs were merged and any references to the Flack parameter were removed.

### Crystal data

**Table:**

C_25_H_34_N_2_O_6_	*F*(000) = 984
*M**_r_* = 458.54	*D*_x_ = 1.259 Mg m^−^^3^
Orthorhombic, *P*2_1_2_1_2_1_	Mo *K*α radiation, λ = 0.71073 Å
Hall symbol: P 2ac 2ab	Cell parameters from 4934 reflections
*a* = 8.0978 (2) Å	θ = 2.4–26.4°
*b* = 10.3660 (3) Å	µ = 0.09 mm^−^^1^
*c* = 28.8194 (8) Å	*T* = 180 K
*V* = 2419.15 (11) Å^3^	Platelet, colourless
*Z* = 4	0.27 × 0.21 × 0.06 mm

### Data collection

**Table:**

Agilent Xcalibur Sapphire1 long-nozzle diffractometer	2540 reflections with *I* > 2σ(*I*)
Radiation source: fine-focus sealed tube	*R*_int_ = 0.034
Graphite monochromator	θ_max_ = 26.4°, θ_min_ = 2.4°
Detector resolution: 8.2632 pixels mm^-1^	*h* = −10→10
ω scan	*k* = −12→12
27441 measured reflections	*l* = −35→36
2829 independent reflections	

### Refinement

**Table:**

Refinement on *F*^2^	Primary atom site location: structure-invariant direct methods
Least-squares matrix: full	Secondary atom site location: difference Fourier map
*R*[*F*^2^ > 2σ(*F*^2^)] = 0.034	Hydrogen site location: inferred from neighbouring sites
*wR*(*F*^2^) = 0.093	H-atom parameters constrained
*S* = 1.05	*w* = 1/[σ^2^(*F*_o_^2^) + (0.0508*P*)^2^ + 0.396*P*] where *P* = (*F*_o_^2^ + 2*F*_c_^2^)/3
2829 reflections	(Δ/σ)_max_ = 0.002
302 parameters	Δρ_max_ = 0.19 e Å^−^^3^
0 restraints	Δρ_min_ = −0.18 e Å^−^^3^

### Special details

**Table:**

Geometry. All s.u.'s (except the s.u. in the dihedral angle between two l.s. planes) are estimated using the full covariance matrix. The cell s.u.'s are taken into account individually in the estimation of s.u.'s in distances, angles and torsion angles; correlations between s.u.'s in cell parameters are only used when they are defined by crystal symmetry. An approximate (isotropic) treatment of cell s.u.'s is used for estimating s.u.'s involving l.s. planes.
Refinement. Refinement of *F*^2^ against ALL reflections. The weighted *R*-factor *wR* and goodness of fit *S* are based on *F*^2^, conventional *R*-factors *R* are based on *F*, with *F* set to zero for negative *F*^2^. The threshold expression of *F*^2^ > 2σ(*F*^2^) is used only for calculating *R*-factors(gt) *etc*. and is not relevant to the choice of reflections for refinement. *R*-factors based on *F*^2^ are statistically about twice as large as those based on *F*, and *R*- factors based on ALL data will be even larger.

### Fractional atomic coordinates and isotropic or equivalent isotropic displacement parameters (Å^2^)

**Table:**

	*x*	*y*	*z*	*U*_iso_*/*U*_eq_	
C1	0.9344 (2)	0.90928 (19)	−0.02860 (7)	0.0367 (4)	
H1	0.8850	0.8325	−0.0428	0.044*	
C2	0.9628 (2)	1.01210 (18)	−0.06365 (7)	0.0365 (4)	
H2	1.0098	1.0913	−0.0506	0.044*	
C3	0.8665 (2)	1.0334 (2)	−0.10613 (7)	0.0396 (4)	
C4	0.8570 (3)	1.1717 (2)	−0.12197 (7)	0.0445 (5)	
H4A	0.8464	1.1734	−0.1555	0.053*	
H4B	0.9593	1.2150	−0.1139	0.053*	
C5	0.7118 (3)	1.2463 (2)	−0.10047 (8)	0.0462 (5)	
H5A	0.7327	1.3380	−0.1037	0.055*	
H5B	0.6123	1.2265	−0.1178	0.055*	
C6	0.6825 (2)	1.21618 (18)	−0.05000 (7)	0.0379 (4)	
H6	0.7828	1.2067	−0.0313	0.045*	
C7	0.5359 (2)	1.1463 (2)	−0.03197 (7)	0.0396 (4)	
C8	0.5440 (2)	1.06794 (19)	0.01276 (7)	0.0394 (4)	
H8	0.4306	1.0443	0.0211	0.047*	
C9	0.6417 (2)	0.94191 (19)	0.00614 (7)	0.0372 (4)	
H9A	0.6198	0.9086	−0.0247	0.045*	
H9B	0.6022	0.8787	0.0283	0.045*	
C10	0.8304 (2)	0.95749 (18)	0.01223 (7)	0.0335 (4)	
H10	0.8530	1.0498	0.0158	0.040*	
C11	1.0873 (3)	0.8737 (2)	0.03774 (8)	0.0446 (5)	
C12	0.9101 (3)	0.8883 (2)	0.05318 (7)	0.0412 (5)	
H12	0.8608	0.8024	0.0561	0.049*	
C13	0.9013 (3)	0.9554 (3)	0.10023 (7)	0.0496 (5)	
H13A	0.9726	0.9101	0.1218	0.060*	
H13B	0.9435	1.0425	0.0970	0.060*	
C14	0.7331 (3)	0.9440 (2)	−0.12263 (8)	0.0483 (5)	
H14A	0.7379	0.9369	−0.1558	0.073*	
H14B	0.6273	0.9775	−0.1136	0.073*	
H14C	0.7486	0.8604	−0.1090	0.073*	
C15	0.3946 (3)	1.1047 (3)	−0.06229 (9)	0.0563 (6)	
H15A	0.2926	1.1346	−0.0492	0.084*	
H15B	0.3928	1.0123	−0.0643	0.084*	
H15C	0.4082	1.1407	−0.0927	0.084*	
C16	0.6773 (3)	0.8326 (2)	0.13338 (8)	0.0527 (6)	
H16A	0.7548	0.7954	0.1553	0.063*	
H16B	0.6741	0.7773	0.1062	0.063*	
C17	0.5082 (3)	0.8371 (2)	0.15515 (8)	0.0536 (6)	
H17A	0.4288	0.8697	0.1328	0.064*	
H17B	0.4746	0.7508	0.1641	0.064*	
C18	0.5649 (4)	1.0502 (2)	0.18283 (8)	0.0589 (6)	
H18A	0.5678	1.1051	0.2101	0.071*	
H18B	0.4873	1.0870	0.1609	0.071*	
C19	0.7342 (4)	1.0444 (2)	0.16120 (8)	0.0562 (6)	
H19A	0.7689	1.1308	0.1526	0.067*	
H19B	0.8125	1.0112	0.1837	0.067*	
C20	0.3698 (3)	0.9137 (3)	0.22451 (7)	0.0543 (6)	
C21	0.2250 (4)	0.8504 (4)	0.21250 (9)	0.0761 (9)	
H21	0.2159	0.8136	0.1832	0.091*	
C22	0.3734 (4)	0.9667 (3)	0.26908 (8)	0.0709 (8)	
C23	0.0932 (4)	0.8409 (5)	0.24322 (11)	0.0987 (13)	
H23	−0.0019	0.7968	0.2345	0.118*	
C24	0.1028 (5)	0.8958 (5)	0.28612 (11)	0.1027 (14)	
H24	0.0137	0.8906	0.3064	0.123*	
C25	0.2443 (5)	0.9589 (4)	0.29923 (10)	0.0941 (12)	
H25	0.2519	0.9960	0.3285	0.113*	
N1	0.7348 (2)	0.96134 (17)	0.11978 (6)	0.0455 (4)	
N2	0.5106 (3)	0.92072 (19)	0.19603 (6)	0.0495 (5)	
O1	0.5493 (2)	1.28526 (14)	−0.02754 (5)	0.0491 (4)	
O2	1.03111 (18)	0.97758 (15)	−0.10802 (5)	0.0469 (4)	
O3	1.09685 (17)	0.88006 (14)	−0.00863 (5)	0.0451 (4)	
O4	1.2079 (2)	0.85660 (19)	0.06106 (7)	0.0647 (5)	
O5	0.6083 (2)	1.14333 (15)	0.04947 (5)	0.0477 (4)	
H5	0.6267	1.0969	0.0719	0.071*	
O6	0.5154 (3)	1.0222 (2)	0.28351 (6)	0.0802 (7)	
H6A	0.5209	1.0960	0.2733	0.120*	

### Atomic displacement parameters (Å^2^)

**Table:**

	*U*^11^	*U*^22^	*U*^33^	*U*^12^	*U*^13^	*U*^23^
C1	0.0321 (9)	0.0299 (9)	0.0479 (11)	0.0032 (8)	−0.0011 (8)	−0.0058 (8)
C2	0.0312 (9)	0.0324 (9)	0.0459 (11)	0.0008 (8)	0.0036 (8)	−0.0072 (8)
C3	0.0390 (10)	0.0366 (10)	0.0432 (10)	0.0039 (9)	0.0050 (9)	−0.0047 (9)
C4	0.0502 (12)	0.0408 (11)	0.0424 (11)	−0.0014 (10)	0.0038 (10)	0.0026 (9)
C5	0.0512 (12)	0.0354 (10)	0.0520 (12)	0.0050 (10)	−0.0030 (10)	0.0036 (9)
C6	0.0368 (9)	0.0273 (9)	0.0495 (11)	0.0038 (8)	−0.0024 (9)	−0.0031 (8)
C7	0.0292 (9)	0.0368 (10)	0.0528 (11)	0.0055 (8)	0.0020 (9)	−0.0069 (9)
C8	0.0286 (9)	0.0387 (11)	0.0510 (11)	−0.0012 (8)	0.0058 (9)	−0.0084 (9)
C9	0.0334 (9)	0.0302 (9)	0.0481 (11)	−0.0046 (8)	0.0032 (8)	−0.0024 (9)
C10	0.0338 (9)	0.0247 (8)	0.0422 (10)	−0.0027 (8)	0.0003 (8)	−0.0024 (8)
C11	0.0449 (11)	0.0295 (10)	0.0595 (14)	0.0035 (9)	−0.0052 (10)	0.0031 (9)
C12	0.0423 (11)	0.0317 (10)	0.0497 (12)	−0.0038 (9)	−0.0032 (9)	0.0042 (9)
C13	0.0526 (12)	0.0487 (12)	0.0476 (12)	−0.0130 (11)	−0.0063 (10)	0.0030 (10)
C14	0.0514 (12)	0.0449 (12)	0.0486 (12)	0.0008 (11)	−0.0045 (10)	−0.0093 (10)
C15	0.0347 (10)	0.0661 (15)	0.0681 (15)	0.0008 (11)	−0.0093 (11)	−0.0049 (13)
C16	0.0673 (15)	0.0400 (11)	0.0507 (13)	−0.0082 (11)	0.0034 (11)	−0.0016 (10)
C17	0.0652 (15)	0.0479 (12)	0.0477 (12)	−0.0124 (12)	0.0016 (11)	−0.0064 (10)
C18	0.0862 (18)	0.0468 (13)	0.0437 (12)	−0.0020 (14)	0.0024 (12)	−0.0055 (10)
C19	0.0812 (17)	0.0421 (12)	0.0453 (12)	−0.0141 (13)	−0.0024 (12)	−0.0038 (10)
C20	0.0631 (14)	0.0615 (15)	0.0383 (11)	0.0001 (12)	−0.0038 (10)	0.0010 (10)
C21	0.0652 (17)	0.109 (2)	0.0540 (15)	−0.0100 (18)	0.0020 (13)	−0.0159 (16)
C22	0.0793 (18)	0.093 (2)	0.0400 (12)	−0.0080 (18)	−0.0029 (13)	−0.0030 (14)
C23	0.0665 (18)	0.152 (4)	0.077 (2)	−0.017 (2)	0.0028 (16)	−0.017 (2)
C24	0.080 (2)	0.166 (4)	0.0622 (18)	−0.005 (3)	0.0191 (17)	−0.009 (2)
C25	0.099 (2)	0.136 (3)	0.0474 (15)	−0.010 (3)	0.0122 (16)	−0.0135 (19)
N1	0.0591 (11)	0.0380 (9)	0.0395 (9)	−0.0088 (9)	−0.0025 (8)	−0.0001 (8)
N2	0.0633 (12)	0.0474 (10)	0.0377 (9)	−0.0045 (9)	−0.0025 (9)	−0.0010 (8)
O1	0.0493 (8)	0.0347 (7)	0.0632 (9)	0.0146 (7)	0.0045 (8)	−0.0050 (7)
O2	0.0422 (8)	0.0502 (9)	0.0483 (8)	0.0074 (7)	0.0107 (7)	−0.0054 (7)
O3	0.0345 (7)	0.0412 (8)	0.0595 (9)	0.0091 (6)	0.0002 (7)	0.0014 (7)
O4	0.0506 (10)	0.0639 (11)	0.0795 (12)	0.0140 (9)	−0.0195 (9)	0.0087 (10)
O5	0.0556 (9)	0.0409 (8)	0.0464 (8)	0.0056 (7)	0.0029 (7)	−0.0096 (7)
O6	0.0937 (15)	0.1145 (18)	0.0323 (8)	−0.0403 (14)	−0.0040 (9)	−0.0159 (10)

### Geometric parameters (Å, º)

**Table:**

C1—O3	1.468 (2)	C13—H13A	0.9700
C1—C2	1.486 (3)	C13—H13B	0.9700
C1—C10	1.531 (3)	C14—H14A	0.9600
C1—H1	0.9800	C14—H14B	0.9600
C2—O2	1.438 (2)	C14—H14C	0.9600
C2—C3	1.468 (3)	C15—H15A	0.9600
C2—H2	0.9800	C15—H15B	0.9600
C3—O2	1.454 (2)	C15—H15C	0.9600
C3—C14	1.501 (3)	C16—N1	1.467 (3)
C3—C4	1.507 (3)	C16—C17	1.507 (4)
C4—C5	1.538 (3)	C16—H16A	0.9700
C4—H4A	0.9700	C16—H16B	0.9700
C4—H4B	0.9700	C17—N2	1.463 (3)
C5—C6	1.507 (3)	C17—H17A	0.9700
C5—H5A	0.9700	C17—H17B	0.9700
C5—H5B	0.9700	C18—N2	1.462 (3)
C6—O1	1.447 (2)	C18—C19	1.507 (4)
C6—C7	1.484 (3)	C18—H18A	0.9700
C6—H6	0.9800	C18—H18B	0.9700
C7—O1	1.450 (3)	C19—N1	1.472 (3)
C7—C15	1.503 (3)	C19—H19A	0.9700
C7—C8	1.525 (3)	C19—H19B	0.9700
C8—O5	1.415 (2)	C20—C21	1.387 (4)
C8—C9	1.539 (3)	C20—C22	1.397 (3)
C8—H8	0.9800	C20—N2	1.407 (3)
C9—C10	1.546 (3)	C21—C23	1.390 (4)
C9—H9A	0.9700	C21—H21	0.9300
C9—H9B	0.9700	C22—O6	1.352 (4)
C10—C12	1.524 (3)	C22—C25	1.362 (4)
C10—H10	0.9800	C23—C24	1.363 (5)
C11—O4	1.198 (3)	C23—H23	0.9300
C11—O3	1.340 (3)	C24—C25	1.372 (5)
C11—C12	1.510 (3)	C24—H24	0.9300
C12—C13	1.525 (3)	C25—H25	0.9300
C12—H12	0.9800	O5—H5	0.8200
C13—N1	1.463 (3)	O6—H6A	0.8200
			
O3—C1—C2	106.00 (15)	N1—C13—H13A	108.8
O3—C1—C10	105.01 (15)	C12—C13—H13A	108.8
C2—C1—C10	111.93 (16)	N1—C13—H13B	108.8
O3—C1—H1	111.2	C12—C13—H13B	108.8
C2—C1—H1	111.2	H13A—C13—H13B	107.7
C10—C1—H1	111.2	C3—C14—H14A	109.5
O2—C2—C3	60.01 (12)	C3—C14—H14B	109.5
O2—C2—C1	119.04 (16)	H14A—C14—H14B	109.5
C3—C2—C1	126.31 (18)	C3—C14—H14C	109.5
O2—C2—H2	113.6	H14A—C14—H14C	109.5
C3—C2—H2	113.6	H14B—C14—H14C	109.5
C1—C2—H2	113.6	C7—C15—H15A	109.5
O2—C3—C2	58.99 (12)	C7—C15—H15B	109.5
O2—C3—C14	113.75 (17)	H15A—C15—H15B	109.5
C2—C3—C14	123.63 (19)	C7—C15—H15C	109.5
O2—C3—C4	114.47 (17)	H15A—C15—H15C	109.5
C2—C3—C4	115.01 (18)	H15B—C15—H15C	109.5
C14—C3—C4	117.03 (19)	N1—C16—C17	111.8 (2)
C3—C4—C5	113.32 (18)	N1—C16—H16A	109.3
C3—C4—H4A	108.9	C17—C16—H16A	109.3
C5—C4—H4A	108.9	N1—C16—H16B	109.3
C3—C4—H4B	108.9	C17—C16—H16B	109.3
C5—C4—H4B	108.9	H16A—C16—H16B	107.9
H4A—C4—H4B	107.7	N2—C17—C16	110.0 (2)
C6—C5—C4	113.91 (18)	N2—C17—H17A	109.7
C6—C5—H5A	108.8	C16—C17—H17A	109.7
C4—C5—H5A	108.8	N2—C17—H17B	109.7
C6—C5—H5B	108.8	C16—C17—H17B	109.7
C4—C5—H5B	108.8	H17A—C17—H17B	108.2
H5A—C5—H5B	107.7	N2—C18—C19	110.2 (2)
O1—C6—C7	59.28 (12)	N2—C18—H18A	109.6
O1—C6—C5	116.54 (17)	C19—C18—H18A	109.6
C7—C6—C5	124.42 (19)	N2—C18—H18B	109.6
O1—C6—H6	114.9	C19—C18—H18B	109.6
C7—C6—H6	114.9	H18A—C18—H18B	108.1
C5—C6—H6	114.9	N1—C19—C18	111.2 (2)
O1—C7—C6	59.10 (13)	N1—C19—H19A	109.4
O1—C7—C15	113.14 (19)	C18—C19—H19A	109.4
C6—C7—C15	123.0 (2)	N1—C19—H19B	109.4
O1—C7—C8	116.85 (17)	C18—C19—H19B	109.4
C6—C7—C8	121.45 (17)	H19A—C19—H19B	108.0
C15—C7—C8	111.79 (19)	C21—C20—C22	115.7 (3)
O5—C8—C7	110.71 (17)	C21—C20—N2	124.3 (2)
O5—C8—C9	111.84 (17)	C22—C20—N2	119.9 (3)
C7—C8—C9	111.69 (16)	C20—C21—C23	121.6 (3)
O5—C8—H8	107.5	C20—C21—H21	119.2
C7—C8—H8	107.5	C23—C21—H21	119.2
C9—C8—H8	107.5	O6—C22—C25	118.8 (3)
C8—C9—C10	113.91 (16)	O6—C22—C20	117.9 (3)
C8—C9—H9A	108.8	C25—C22—C20	123.1 (3)
C10—C9—H9A	108.8	C24—C23—C21	120.3 (3)
C8—C9—H9B	108.8	C24—C23—H23	119.9
C10—C9—H9B	108.8	C21—C23—H23	119.9
H9A—C9—H9B	107.7	C23—C24—C25	119.7 (3)
C12—C10—C1	102.06 (15)	C23—C24—H24	120.1
C12—C10—C9	117.19 (17)	C25—C24—H24	120.1
C1—C10—C9	114.97 (16)	C22—C25—C24	119.6 (3)
C12—C10—H10	107.3	C22—C25—H25	120.2
C1—C10—H10	107.3	C24—C25—H25	120.2
C9—C10—H10	107.3	C13—N1—C16	110.91 (19)
O4—C11—O3	121.3 (2)	C13—N1—C19	109.86 (18)
O4—C11—C12	128.6 (2)	C16—N1—C19	108.35 (16)
O3—C11—C12	110.09 (18)	C20—N2—C18	116.3 (2)
C11—C12—C10	102.80 (17)	C20—N2—C17	115.4 (2)
C11—C12—C13	110.61 (18)	C18—N2—C17	109.78 (17)
C10—C12—C13	117.03 (17)	C6—O1—C7	61.63 (12)
C11—C12—H12	108.7	C2—O2—C3	61.00 (13)
C10—C12—H12	108.7	C11—O3—C1	110.46 (16)
C13—C12—H12	108.7	C8—O5—H5	109.5
N1—C13—C12	113.86 (18)	C22—O6—H6A	109.5

### Hydrogen-bond geometry (Å, º)

**Table:**

*D*—H···*A*	*D*—H	H···*A*	*D*···*A*	*D*—H···*A*
O5—H5···N1	0.82	2.15	2.952 (2)	164
C1—H1···O4^i^	0.98	2.49	3.440 (3)	165
C10—H10···O1^ii^	0.98	2.36	3.232 (2)	148
C18—H18*A*···O6	0.97	2.32	2.943 (3)	121
C25—H25···O2^iii^	0.93	2.55	3.299 (4)	138

Symmetry codes: (i) *x*−1/2, −*y*+3/2, −*z*; (ii) *x*+1/2, −*y*+5/2, −*z*; (iii) −*x*+3/2, −*y*+2, *z*+1/2.
